# Development and Evaluation of a Sound-Swapped Video Database for Misophonia

**DOI:** 10.3389/fpsyg.2022.890829

**Published:** 2022-07-22

**Authors:** Patrawat Samermit, Michael Young, Allison K. Allen, Hannah Trillo, Sandhya Shankar, Abigail Klein, Chris Kay, Ghazaleh Mahzouni, Veda Reddy, Veronica Hamilton, Nicolas Davidenko

**Affiliations:** High Level Perception Laboratory, Department of Psychology, University of California, Santa Cruz, Santa Cruz, CA, United States

**Keywords:** misophonia, aversive sounds, trigger sounds, stimuli development, stimuli validation, video database, multimodal integration, multimodal perception

## Abstract

Misophonia has been characterized as intense negative reactions to specific trigger sounds (often orofacial sounds like chewing, sniffling, or slurping). However, recent research suggests high-level, contextual, and multisensory factors are also involved. We recently demonstrated that neurotypicals’ negative reactions to aversive sounds (e.g., nails scratching a chalkboard) are attenuated when the sounds are synced with *positive attributable video sources* (PAVS; e.g., tearing a piece of paper). To assess whether this effect generalizes to misophonic triggers, we developed a Sound-Swapped Video (SSV) database for use in misophonia research. In Study 1, we created a set of 39 video clips depicting common trigger sounds (*original video sources*, OVS) and a corresponding set of 39 PAVS temporally synchronized with the OVS videos. In Study 2, participants (*N* = 34) rated the 39 PAVS videos for their audiovisual match and pleasantness. We selected the 20 PAVS videos with best match scores for use in Study 3. In Study 3, a new group of participants (*n* = 102) observed the 20 selected PAVS and 20 corresponding OVS and judged the pleasantness or unpleasantness of each sound in the two contexts accompanying each video. Afterward, participants completed the Misophonia Questionnaire (MQ). The results of Study 3 show a robust attenuating effect of PAVS videos on the reported unpleasantness of trigger sounds: trigger sounds were rated as significantly less unpleasant when paired with PAVS with than OVS. Moreover, this attenuating effect was present in nearly every participant (99 out of 102) regardless of their score on the MQ. In fact, we found a moderate positive correlation between the PAVS-OVS difference and misophonia severity scores. Overall our results provide validation that the SSV database is a useful stimulus database to study how misophonic responses can be modulated by visual contexts. Here, we release the SSV database with the best 18 PAVS and 18 OVS videos used in Study 3 along with aggregate ratings of audio-video match and pleasantness (https://osf.io/3ysfh/). We also provide detailed instructions on how to produce these videos, with the hope that this database grows and improves through collaborations with the community of misophonia researchers.

## Introduction

In the early 2000’s, Jastreboff and Jastreboff (2001) coined *misophonia* as “the hatred of sound,” where some individuals have intense emotional and physical reactions to specific trigger sounds. Everyday sounds, such as chewing, breathing, drinking, nasal sounds, or finger tapping, can act as triggers to people with misophonia (Edelstein et al., 2013; Wu et al., 2014). Although many people might find the sound of slurping at the dinner table rude or annoying, individuals with misophonia may respond to that trigger sound with extreme, immediate emotional, physical, behavioral, and cognitive responses ranging from feelings of disgust, anxiety, and anger to an uncontrollable desire to physically harm the person producing it (Edelstein et al., 2013; Wu et al., 2014; Swedo et al., 2021). The misophonic trigger reaction is one of heightened autonomic arousal and physiological responses, such as tightened muscles or pressure in one’s chest, arms, head, or across the body (Edelstein et al., 2013; Brout et al., 2018). At times, individuals with misophonia may suffer functional impediments in their occupational, academic, and social lives (Schröder et al., 2013; Webber and Storch, 2015) as a result of their emotional, cognitive, physiological, or behavioral responses to trigger sounds, including avoiding or leaving situations.

Misophonia was initially considered to be an audiological disorder, but recent research has found that individuals’ trigger reactions are not tied to specific physical characteristics of the sound like pitch, timbre, or volume (Jastreboff and Jastreboff, 2014). More recently, interdisciplinary perspectives have brought to light a more holistic understanding of the misophonic experience. Recently, a cohort of experts has established a consensus definition for misophonia that takes these different factors into consideration. According to Swedo et al. (2021), misophonia is “a disorder of decreased tolerance to specific sounds or stimuli associated with these sounds” associated with “the specific pattern or meaning to an individual” (p. 22). The misophonic trigger response is idiosyncratic and tied to individual differences (Brout et al., 2018), with growing evidence that contextual factors, such as an individual’s perceived level of control, the context where a trigger stimulus is experienced, and the interpersonal relationships involved, all modulate the trigger response (Edelstein et al., 2020; Swedo et al., 2021).

Importantly, the trigger response may be influenced by higher-order contexts learned alongside trigger sounds. Edelstein et al. (2020) found that contextual information that is presented alongside a sound can change the way it is perceived. In their experiment, they asked control participants and participants with misophonia to rate the aversiveness of sounds from three categories: human eating sounds (a common misophonic trigger), animal eating sounds, or no eating sounds. They found that participants with misophonia rated human eating sounds *that they incorrectly identified as animal eating sounds* or as non-eating sounds as less aversive compared to when they correctly identified them as human eating sounds. Their findings suggest that the attributed source of a sound influences its perceived aversiveness.

Neuroimaging work by Kumar et al. (2017) found that when individuals with misophonia perceive trigger sounds, their anterior insular cortex has increased activity, resulting in heightened salience and emotional response and interoception in response to trigger sounds. However, in more recent work, Kumar et al. (2021) propose that this increased response is not a response to sound itself, but a result of “hyper-mirroring” in higher-order motor systems tied to the perception and production of trigger sounds. They found that compared to controls, the misophonia group had stronger connectivity between (1) the auditory, visual, and ventral premotor cortex responsible for orofacial movements, (2) the auditory cortex and orofacial motor areas during sound perception generally, and (3) stronger activation in the orofacial motor area in response to trigger sounds. They propose the sound and visual cues tied to the sound are not the cause of increased responses. Rather, they are the medium through which the motor action of a sound-maker is mirrored by an individual with misophonia, driving increased arousal responses. Their findings implicate the mirror neuron system in the experience of misophonia. When an individual with misophonia hears or sees another person doing a triggering action, auditory and visual mirroring processes allow them to create a representation of these actions (Rizzolatti et al., 1996; Aglioti and Pazzaglia, 2010), including their behavioral intentions in social interactions (Gallese, 2009). The motor basis for misophonia supports the hypothesis that high-level contexts, including the interpretation of social intent of the trigger producer, may play a role in the experience of misophonic trigger responses.

As such, we hypothesize that individuals with misophonia may have learned negative associations between trigger sounds and their source, which are predominantly orofacial sounds produced by others during eating or during repetitive movements (Jager et al., 2020). We propose that if we can disrupt this association by providing a plausible alternative visual source, individuals with misophonia may experience an alleviated misophonic trigger response. Past research supports the role that visual capture has on the experience of sounds. Cox (2008) found that the concurrent presentation of an image associated with a horrible sound resulted in participants perceiving the sound more horribly than when it was presented with an unassociated or control image. Thus, a new association provided by static visual cues affected people’s response to horrible sounds. Moreover, we know that visual-auditory integration can be strengthened by *temporal synchronization*, such as in the *McGurk effect*: making ambiguous auditory information like phonemes differentially discernable depending on mouth shape paired with it (/ba/perceived as/da/when participants view lips creating/ga/; McGurk and MacDonald, 1976).

Recently, Samermit et al. (2019) expanded on the role of synchronized audio-visual integration in the perception of aversive sounds. In that study, we presented neurotypical observers with a set of aversive sounds (e.g., sound of nails scratching on a chalkboard) synced with either the *Original Video Source* (OVS; e.g., a video of someone dragging nails down a chalkboard) or a *Positive Alternative Video Source* (PAVS; e.g., a video of someone playing the flute). Participants provided ratings of *discomfort* (how comfortable or uncomfortable the sound made them feel), *unpleasantness* (how pleasant or unpleasant the sound was), and *bodily sensations* (the intensity of any experienced physiological response elicited by the sound). Across all three measures, we found that the cross-sensory temporal syncing of aversive sounds to positive alternative video sources (PAVS) attenuated the negative responses compared to the presentation of sounds with the original video source (OVS).

We hypothesize that the findings of Samermit et al. (2019) will extend to modulate misophonic trigger reactions: pairing a misophonic trigger sound with a PAVS will reduce the negative response to the sound. Testing this hypothesis experimentally required us to develop a novel database of misophonic trigger stimuli: Trigger sounds along with their OVS that produced the trigger sound, and the same trigger sounds synched with PAVS that could feasibly have produced those sounds. In order for the temporal synchronization of visual and auditory cues to have any potential effect on misophonic trigger reactions, we needed to ensure that our stimuli (1) had well-matched audio and visual synchronization, and (2) that the PAVS were actually perceived more positively than OVS.

Here we present our 3-part methodology for the development and evaluation of a *Sound-Swapped Video* (SSV) database for misophonia, which we release for use in research. The database includes 20 pairs of PAVS and OVS videos along with the original trigger sound audio files, and aggregate ratings of each of the stimuli. In Study 1, we present the process we developed to generate and evaluate these audio-visual stimuli.

In Study 1, we conducted an idea-generation study to identify examples of alternative videos to create. In Study 2, we present the validation of 39 PAVS stimuli to identify a subset of stimuli that are perceived as relatively pleasant and have adequate audio-video match. In Study 3, we presented individuals with the 20 best PAVS and their associated OVS, to identify whether trigger sounds paired with PAVS are perceived as more pleasant than the same sounds paired with OVS. Although we recruited a general population for these studies, we collected responses on Misophonia Questionnaire (MQ; Wu et al., 2014) allowing us to relate participants’ responses to these sounds to their self-reported sensitivity to misophonic triggers.

The validated Sound-Swapped Video (SSV) database for misophonia is available on OSF^1^ for use and collaboration by misophonia researchers.

## Materials and Methods

Here we present the methodologies for Study 1 (Generation and Evaluation of Audio-Visual Stimuli), Study 2 (Evaluation of 39 PAVS stimuli), and Study 3 (Evaluation of the best 20 PAVS and 20 OVS stimuli).

### Study 1: Generation and Evaluation of Audio-Visual Stimuli

#### Stimuli

Based on an analysis of the 80 interviews, we generated a list of commonly reported trigger sounds and grouped them into the following ten categories: crunchy chewing, wet chewing, slurping, swishing, sniffling, gulping, drumming, scraping, clicking, and squeaking.

#### Interview Process

We identified each individual’s top three triggers from a set of semi-structured interviews with 80 participants with misophonia. These interviews were part of a longer-term project to explore how different high-level contexts such as one’s social environment and social interactions, attention, visual cues, or experience of agency and control may be related to participants’ misophonic responses. As such, these interviews were designed to be idiographic (Molenaar, 2004; Barlow and Nock, 2009) and understand in-depth an individual participant’s relationship with their misophonic trigger sounds and reactions.

Participants were recruited from the greater Bay Area, California and Santa Cruz, California through the psychology department’s undergraduate participant pool, by word of mouth, and through the use of recruitment flyers on social media. Prior to the core interview, participants completed a pre-screen phone call with one of the researchers to confirm their experiences with trigger sounds were consistent with existing descriptions of misophonic trigger reactions.

Before participating in the interview, participants completed a consent form and the Misophonia Questionnaire (MQ; Wu et al., 2014). The MQ consists of three sections, including the *Misophonia Symptom Scale* (where participants were asked to rate how sensitive they are to a category of sound compared to other people), the *Misophonia Emotions and Behaviors Scale* (where participants rate their reactions associated with misophonia symptoms), and the *Misophonia Severity Scale*. The first section, the Misophonia Symptom Scale, consists of 7 items, where participants can indicate specific sound sensitivities. Participants respond to items like “Nasal sounds” or “People eating” between 0 (Not at all True) to 4 (Always True). The second section, the Misophonia Emotions and Behaviors Scale, assesses emotional and behavioral responses to trigger sounds, and consists of 10 items (e.g., “physically aggressive” or “leave environment”) with the same response scale as section one. These first two sections are summed into a total MQ Sensitivity Score, which ranges from 0 to 68 points.

The final section, the Misophonia Severity Scale, consists of a single question where participants rate the severity of their sensitivity from 0 (minimal) to 15 (very severe). A score above 7 on this scale indicates clinically significant misophonic reactions. For participants in this interview about their misophonic experiences, the mean Misophonia Sensitivity Score (combined score on the first two sections; maximum 68) was 36.1 (*SD*: 10.5) and the mean Severity Score (maximum 15) was 5.5 (*SD*: 2.2). This is consistent with existing research where Wu et al. (2014) found a clinical population had a mean Misophonia Sensitivity Score of 31.21 (*SD*: 7.64), and Zhou et al. (2017) found a clinical population of students in China had a mean Misophonia Sensitivity Score of 33.1 (*SD*: 10.73). In the current study, the internal consistency (Cronbach’s alpha) was 0.715 for the Misophonia Symptom Scale, 0.838 for the Misophonia Emotions and Behaviors Scale, and 0.843 for the Total score (the combination of these two parts).

The interview examined participants’ experiences with misophonia including questions on their trigger sounds and trigger reactions, the relationship between social experiences and their trigger reactions, and how other multimodal experiences, such as seeing the source of a sound, may be related to their trigger reactions. Each semi-structured interview was conducted on Zoom with an average length of 65 min. Participants were asked 32 primary questions and were asked follow up questions at the interviewer’s discretion. The interview was split into six sections:

1. Characterization of trigger sounds and trigger reactions, e.g., “What are your 3 worst trigger sounds? Why are they the worst?”
2. Personal and family history of misophonic experiences, e.g., “How old were you when you first experienced a misophonic response to a sound?”
3. Contextualizing trigger reactions, e.g., “Is there anything that makes your trigger sounds more tolerable for you? If so, how/why?”
4. Top-down effects and contexts associated with trigger reactions, e.g., “Has there ever been a time when the presence of trigger sounds affected your ability to focus on your goals?”).
5. Dynamic factors associated with trigger reactions, e.g., “Do you notice any difference in how you react to trigger sounds when you haven’t slept enough?”
6. Multisensory experiences similar to trigger reactions, e.g., “Have you ever SEEN anything that makes you feel the same as when you hear a trigger sound?”

We categorized the top 3 trigger sounds into high level categories, and used these categories as seed ideas for our stimuli.

Video generation process: For each trigger category, we constructed a number of audiovisual stimuli including OVS of the triggers (e.g., a video recording of a person chewing chips) and PAVS of the triggers (e.g., a video recording of a person tearing a piece of paper, in sync with the sound of chewing chips). This was done in 4 stages, which we describe below. A more detailed manual with step-by-step instructions is available here: (see text footnote 2).

#### Stage 1: Generating Ideas for Positive Attributable Video Sources

To generate ideas for what alternative sources might map well with each trigger sound, we used a combination of two approaches: (1) brainstorming sessions among the researchers and (2) collecting behavioral responses from naïve participants to the sounds. In the brainstorming sessions, researchers listened to or talked about the categories of trigger sounds determined from participants’ interviews, such as slurping or crunchy chewing, and imagined alternative sources that might create a similar sound. For example, for the sound of someone slurping, alternative sources included shuffling a deck of cards, flipping through pages of a book, and raising blinds.

One limitation to the brainstorming session was the possibility of functional fixation (Maier, 1931; Duncker and Lees, 1945) on the part of the researchers, who had prior knowledge about the true source of the sound. To overcome this, we conducted a remote behavioral study that presented 16 naïve participants with several 3-second-long audio clips of trigger sounds, such as the sound of someone slurping or chewing something crunchy, and asked them to try to identify each sound. The sounds and questionnaire were presented using an online survey platform, and participants documented what they thought the sound could be in an open-ended text box. We examined *incorrect guesses* as potential candidates for alternative sources as they were reasonably mistaken for the source sound. For example, the sound of finger drumming was once misidentified as “a rubber ball rapidly bouncing on the floor” and as “a plastic bottle rolling on a desk.”

#### Stage 2: Video-Recording the Positive Attributable Video Sources

The idea generation stage led to a collection of about 3–5 ideas of alternative sources for each of the ten trigger sound categories. Two research assistants then began the process of constructing each pair of PAVS and OVS stimuli.

The first step was to record a roughly 15-second audio-video clip of the Positive Attributable Video Source (PAVS). The reason that the PAVS was recorded first is that since most triggers are human-made orofacial sounds, it is relatively easy to generate those sounds to match the rhythm of a pre-existing PAVS. In contrast, we found it was more difficult to generate a PAVS to match the rhythm of a pre-existing OVS. For instance, it was easier to produce chewing sounds in the rhythm of a person walking on snow, compared to trying to walk in the rhythm of a person chewing. Our process is similar to Foley Sound Design^2^, where sound designers will watch footage from a TV show or movie and produce sound effects post-production in sync with what is occurring visually. In this case, it is the reverse—we produce videos that match up with an existing sound.

PAVS were self-recorded by research assistants with the use of a tripod and a smartphone with an auxiliary shotgun microphone that directly targets the sound of the action and reduces unwanted low gain background noise. Smartphones with similar video capabilities and resolution were used to capture the action of both the PAVS and the OVS stimuli. Most PAVS stimuli involved an agent (for instance a person hammering a stake into the ground or walking on snow), while some PAVS stimuli involved agent-less environmental sources, such as water running down a creek.

#### Stage 3: Recording the Original Video Sources

The next step was to record the OVS stimulus, which always involved a human actor/agent. OVS videos were also self-recorded by research assistants with the use of a tripod and a smartphone with an auxiliary shotgun microphone. The goal of the OVS recording was to create something roughly synchronized to the already recorded PAVS and capture the trigger sound clearly. Therefore the OVS recording process involved the actor attempting to synchronize the trigger action (e.g., chewing) while carefully watching and listening to the previously recorded PAVS (e.g., walking). To aid in this synchronization, the PAVS audio was played through headphones while the video was displayed in Adobe Premiere, allowing the actor to use both visual and auditory cues to determine the rhythm of the to-be-produced OVS. Special consideration was given to recording the audio to match the cadence and dynamics to the video component of the PAVS. The camera was often focused on the orofacial action, which featured the lower half or full face of the human agent. After a successful attempt at creating an OVS, both videos were evaluated to check for the viability of the temporal match. If the video pair was reasonably well synchronized, the raw audio and video were cataloged for further editing. Otherwise, the OVS recording process continued until a good match was achieved.

#### Stage 4: Audio Editing and Normalization

Before the audio was edited, the OVS and PAVS videos were roughly matched on a timeline in Adobe Premiere. A 12-second clip was then selected representing the best matched section that excludes loud noises associated with the beginning and ending of the original recordings. The audio components of the OVS and PAVS were then exported to Audition where the clips were normalized to −3 db. This step raised the volume of quiet parts and lowered the volume of louder parts of the audio waveforms. In some circumstances, additional distracting noises were removed from the waveform with dynamics processing to lower amplitudes of specific frequencies, for instance, a low frequency air conditioning noise that added white noise to the video. This tool was used sparingly as it could end up removing frequencies that are essential to the sound of the OVS.

#### Stage 5: Video Editing

With the audio now normalized, the corresponding video tracks were edited in Adobe Premiere. Due to the prior audio editing and video recording process, the audio waveforms should already be aligned and similar in amplitude, frequency, and height. The two normalized audio and video pairs were then placed on the timeline to find the best fit. The PAVS video was then overdubbed with the OVS sound. However, after an initial playback, additional unwanted sounds may need to be edited out from the audio that affect the believability of the audio-video match. The sounds that were generally removed were not representative of the OVS triggers themselves. For instance, distracting breathing sounds may be spliced out of a chewing sound since they might affect how plausible the match will turn out with PAVS. Additionally, some sections of the OVS audio may be slowed down (up to 85%) or sped up (up to 120%) to create a better match with the PAVS video.

Once these 5 stages were complete, we ended up with two audiovisual stimuli corresponding to a particular trigger sound: the OVS with the original triggering audio and video, and the PAVS with a positive attributable video source dubbed with the triggering OVS sound. In addition, we also cataloged the original sound files as well as the PAVS with its original (non-trigger) sound for potential use in future work.

### Study 2: Evaluation of 39 Positive Attributable Video Sources Stimuli

#### Stimuli

The stimuli for Study 2 were the 39 PAVS stimuli constructed as described above. The stimuli contained non-trigger video sources (e.g., someone stepping on snow) paired with trigger sounds (e.g., chewing). The 39 trigger sounds included several examples of the ten categories described earlier.

#### Participants

We recruited 34 naïve participants (26 women, 6 men; ages 18–28) from the University of California Santa Cruz participant pool who received course credit for their participation. Participants completed a questionnaire *via* Qualtrics where they watched each of the 39 12-second PAVS stimuli in a random order.

#### Procedure

After watching each video, participants provided ratings of how pleasant or unpleasant the video clip was, and how well the sound and video matched. The pleasantness scale used the following response scale: 1 (Very unpleasant), 2 (Somewhat unpleasant), 3 (Neither pleasant nor unpleasant), 4 (Somewhat pleasant), or 5 (Very pleasant). The sound-video match question used the following response scale: 1 (Not a good match), 2 (Slightly good match), 3 (Moderately good match), 4 (Very good match), or 5 (Extremely good match).

### Study 3: Evaluation of the Best 20 Positive Attributable Video Sources and 20 Original Video Sources Stimuli

#### Stimuli

Based on the results of Study 2, we selected the best 20 PAVS stimuli based on the reported quality of the sound-video match, along with the corresponding 20 OVS stimuli. The resulting 40 stimuli were presented to a new group of observers in two possible pseudo-random orders. In both presentation orders, half of the trigger sounds appeared once in the first half and once in the second half, paired with either a PAVS or an OVS video source. The counterbalancing allowed us to collect ratings for each PAVS-OVS pair in two different orders across participants. For half of the participants, “odd” PAVS videos and “even” OVS videos were played first, and “even” PAVS videos and “odd” OVS videos were played second. For the other half of the participants, it was the other way around. Having these two presentation orders allowed us to analyze responses by order (i.e., by whether the PAVS video was presented before or after the OVS video).

#### Participants

We recruited 102 naïve participants (65 women, 33 men, and 4 non-binary; ages 18–29) from the University of California, Santa Cruz Psychology participant pool who received course credit for their participation. Participants completed a questionnaire *via* Qualtrics where they watched and rated the 20 PAVS and 20 OVS stimuli.

#### Procedure

After watching each video, participants provided two ratings as in Study 2, indicating how pleasant or unpleasant the sound was, and how well the sound and video matched, using the same 5-point scales as in Study 2. The primary difference here is that in Study 3 we asked about the pleasantness or unpleasantness of the sound itself, whereas in Study 2, we asked about the pleasantness or unpleasantness of the PAVS video clip as a whole. After viewing and rating all 40 stimuli, participants then completed the Misophonia Questionnaire (MQ) and answered several demographic questions.

## Results

### Study 1

We produced 39 pairs of PAVS/OVS stimuli, resulting in 78 audio-video files in total. Based on the results of Studies 2 and 3, we release a subset of the best 18 PAVS and the corresponding 18 OVS (in folders titled “PAVS videos” and “OVS videos,” respectively) as open-source downloads *via* OSF (see text footnote 2).

### Study 2

**FIGURE 1:**
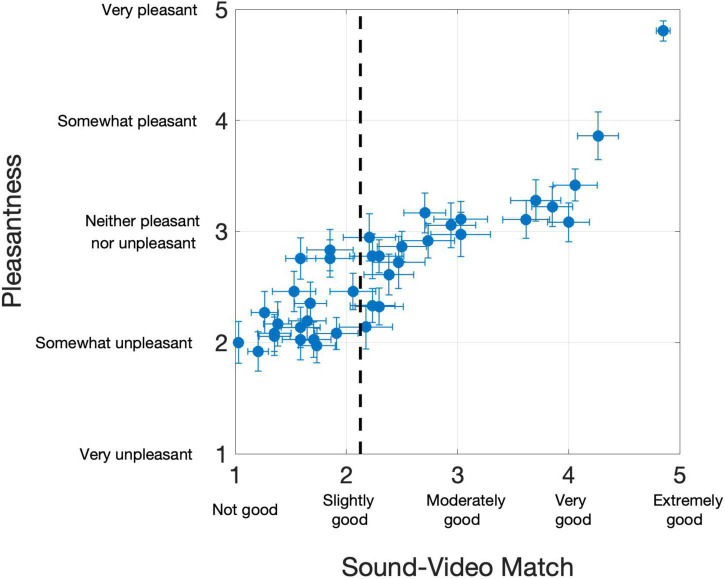
Results from 34 participants in Study 2. Each dot represents a PAVS video. The x-position shows the mean sound-video match rating, and y-position shows the mean pleasantness rating. Horizontal and vertical error bars represent one standard error from the mean on the two scales, respectively. The dotted line represents the sound-video match cut-off we used to select the top 20 stimuli for Study 3.

Overall, the 39 PAVS videos received mean ratings of 2.36 (*SD*: 0.96) on the 5-point sound-video match scale, and 2.67 (*SD*: 0.60) on the pleasantness scale, although there were large differences across the videos Each of the PAVS video’s mean ratings for sound-video match and pleasantness across 34 observers are shown in Figure 1.

Overall, participants provided a wide range of sound-video match ratings of the PAVS videos. Many stimuli ended up with ratings of “slightly good” or below, making them unviable for further use. Many of these videos were excluded due to the video and audio being off sync or, more commonly, due to perceptual differences between the sound quality (timbre) and the material from the visual source (e.g., a hollow bouncing sound could not reasonably originate from a ball lightly hitting a shag carpeted floor). For Study 2, we selected the best 20 PAVS videos based on the sound-video match rating, with a cutoff value of 2.15 in the 5-point sound-video match scale.

### Study 3

**FIGURE 2:**
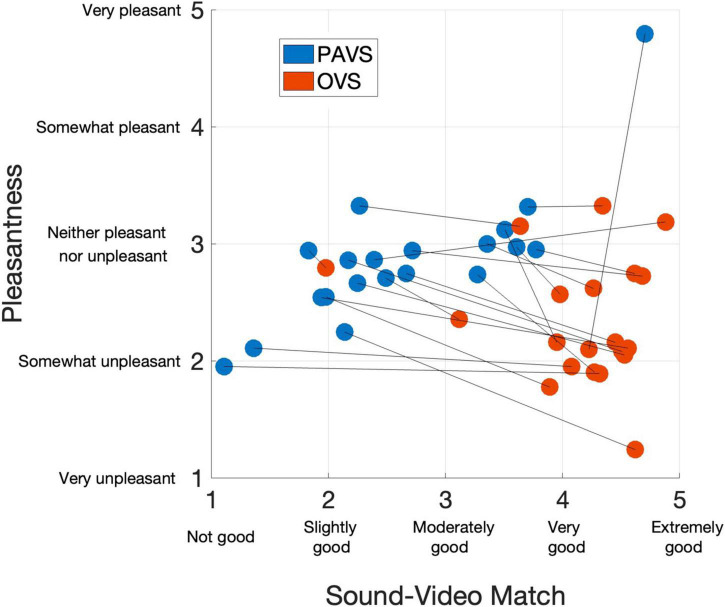
Mean ratings of the 40 stimuli in Study 3 for audio-visual match (x-axis) and pleasantness (y-axis), averaged across 102 observers. Blue dots show PAVS stimuli and red dots show OVS stimuli. Line segments connect the corresponding PAVS and OVS stimuli.

We first examined ratings of the audio-visual match of the 20 PAVS and 20 OVS stimuli. Match ratings for the PAVS videos closely mirrored the results from Study 2 restricted to the best 20 stimuli. The mean rating of sound-video match across the 20 PAVS stimuli was 2.66 (*SD*: 0.91). As expected, match ratings for PAVS videos were consistently lower than those for corresponding OVS videos (mean: 4.15, *SD*: 0.65). Nevertheless a majority (15 of 20) of the PAVS stimuli obtained match ratings of slightly good or above. Figure 2 shows match ratings for all 40 stimuli (x-axis) plotted along with their mean pleasantness ratings (y-axis).

**FIGURE 3:**
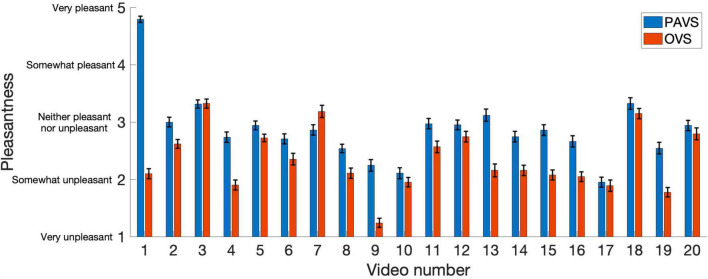
Mean ratings of PAVS and OVS videos averaged across 102 observers. The x-axis represents the video number (arbitrarily assigned) and y-axis represents the 5-point pleasantness scales. PAVS ratings shown in blue and OVS ratings in red. A table shows the content of each of the 20 sounds. Error bars represent the standard error of the mean across participants.

**FIGURE 4:**
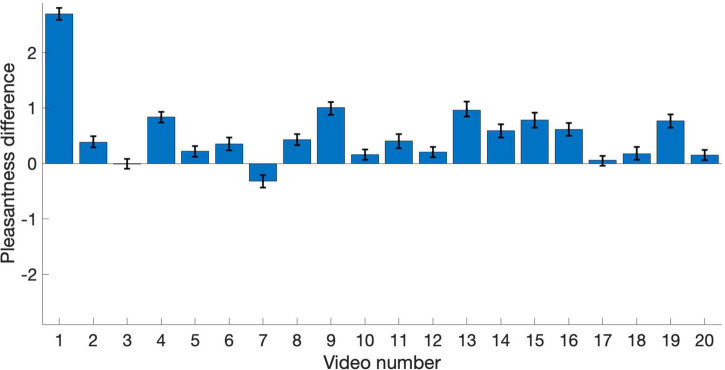
Pleasantness difference scores (PAVS rating minus OVS rating) for the 20 sounds. Each bar represents the average difference score across participants for each video. Error bars represent the standard error of the mean across participants.

**FIGURE 5:**
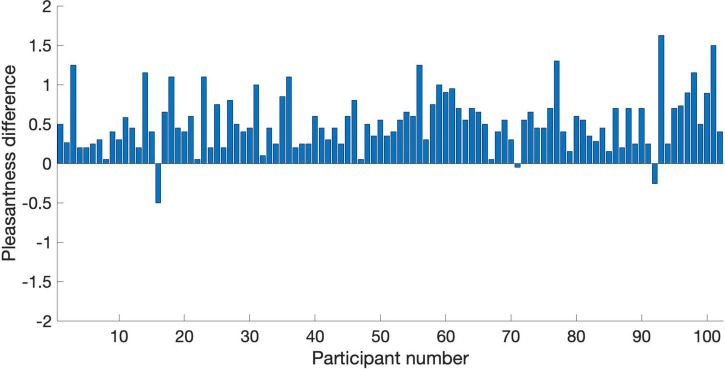
Pleasantness difference scores (PAVS rating minus OVS rating) for the 102 participants in Study 3. Each bar represents the average difference score across the 20 PAVS and 20 OVS for each participant.

The remaining analyses focus on pleasantness ratings. To better visualize the relationship between pleasantness of PAVS and OVS videos, the average pleasantness ratings for the 20 PAVS and 20 corresponding OVS stimuli are shown in Figure 3. For most stimuli (18 out of 20), pleasantness ratings for PAVS-paired sounds were higher than for OVS-paired sounds. A paired *t*-test (*t*_19_ = 3.78, *p* = 0.0013) confirmed this was statistically significant. The difference scores (mean PAVS rating minus mean OVS rating) for the 20 sounds are shown in Figure 4. The mean difference score across videos was 0.52 [95% *CI*: (0.233, 0.812); Cohen’s *d* = 0.845] which represents a large effect, where the maximum difference possible in the 5-point scale was 4. An observer-based analysis confirmed that this effect was nearly universal across our 102 participants. Figure 5 shows this difference score, averaged across the 20 sounds, separately for each participant.Overall, 99 out of 102 reported numerically higher pleasantness ratings of the PAVS-paired sounds compared to the OVS-paired sounds. A paired *t*-test (*t*_101_ = 14.87, *p* < 0.0001) confirmed this effect was significant. The mean difference score across observers was 0.52 [95% *CI*: (0.452, 0.592); Cohen’s *d* = 1.47], representing a very large effect size.

**FIGURE 6:**
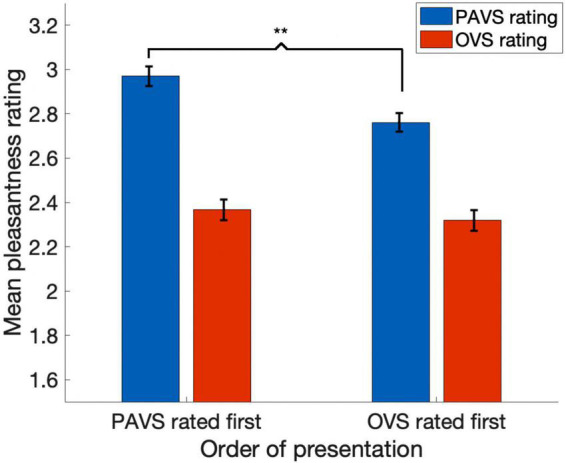
The effect of order of presentation on pleasantness ratings. Blue bars show mean pleasantness ratings for PAVS videos and red bars show mean pleasantness ratings for OVS-paired sounds. The first pair of bars shows results for sounds that were rated in the PAVS context first and OVS context second; the second pair of bars shows results for sounds that were rated in the OVS context first and PAVS context second. Error bars denote one standard error of the mean across 102 observers. The asterisks indicate a significant difference (*p* < 0.0001).

**FIGURE 7:**
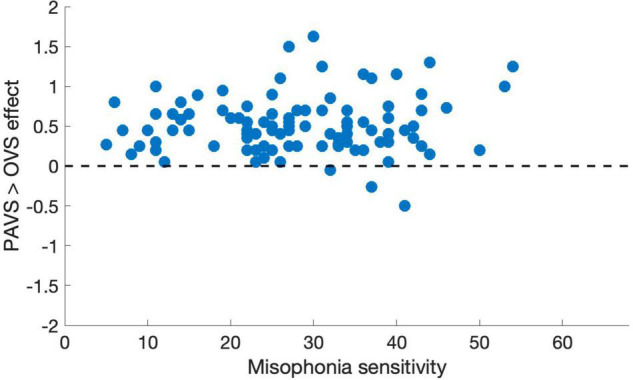
Mean pleasantness difference score for each individual in Study 3, plotted as a function of their total MQ sensitivity score (0–68). Total MQ sensitivity is the sum of an individual’s total for the misophonia symptom scale and the misophonia emotions and behaviors scale.

**FIGURE 8:**
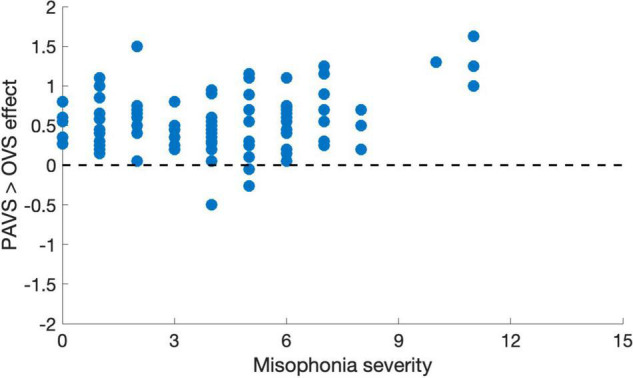
Mean pleasantness difference score for each individual in Study 3, plotted as a function of their MQ severity score (0–15).

Based on the two orders of stimulus presentations across observers that we described earlier, we were able to measure whether the order of presentation (PAVS-first or OVS-first) made a difference in the mean pleasantness ratings of the corresponding sounds. The set of mean pleasantness ratings for PAVS and OVS videos based on whether they were shown first or second are shown in Figure 6. Results show that the order of presentation made a substantial difference. Specifically, PAVS-paired sounds that were first shown in the PAVS context received significantly higher pleasantness ratings (mean = 2.97) compared to PAVS-paired sounds that were first shown in the OVS context (mean = 2.76; *t*_101_ = 5.15, *p* < 0.0001). This presentation order effect was not observed for OVS-paired sounds, which were rated similarly whether they were presented in the OVS context first (mean = 2.37) or in the OVS context second (2.32; *t*_101_ = 1.20, *p* > 0.2).Finally, we examined whether pleasantness ratings varied as a function of individuals’ score on the MQ (Wu et al., 2014). For participants in this study, the mean combined score on the first two sections of the MQ was 27.5 (*SD*: 11.0) and the mean score for the third section was 4.0 (*SD*: 2.6). In this study, the internal consistency (Cronbach’s alpha) was 0.792 for the Misophonia Symptom Scale, 0.834 for the Misophonia Emotions and Behaviors Scale, and 0.863 for the Total score (the combination of these two parts). Figure 7 shows mean pleasantness difference scores plotted as a function of individuals’ Misophonia sensitivity score, which is the total of the misophonia symptom scale and emotions and behavior scale, for a max total of 68. Figure 8 shows the pleasantness ratings as a function of individuals’ Misophonia severity score (based on the final question of the MQ), which asks participants to rate the severity of their sound sensitivity on a scale from 1 (minimal) to 15 (very severe). For this scale, a score of 7 or above would constitute clinically significant misophonic reactions. There are 14 participants out of 101 (13.7% of the sample) that had misophonia severity scores of 7 or above. Participants with high severity [score of 7 or above; mean difference = 0.83, 95% *CI*: (0.58, 1.09)] have significantly larger difference scores compared to those with low severity scores [score of 6 or less; mean difference = 0.47, 95% *CI*: (0.41, 0.54); two-sampled *t*_101_ = 3.76, *p* = 0.0003]. As can be seen in these figures, pleasantness difference scores were positive across most individuals regardless of the sensitivity or severity of their MQ scores. Correlation analyses revealed no relationship between MQ sensitivity and mean difference score (*r* = 0.094, *p* > 0.3), and a moderate positive relationship between MQ severity and mean difference score (*r* = 0.28, *p* = 0.004). Here, participants with higher MQ severity scores showed a significantly *larger* difference in pleasantness ratings of PAVS-paired vs. OVS-paired sounds compared to other participants. Separate analyses of PAVS and OVS ratings revealed that this relationship was driven by individuals with higher severity scores rating OVS as more unpleasant than individuals with lower severity scores; there was no difference between how these groups rated the PAVS-paired sounds. However, we found that the correlation is driven by four participants with very high scores (10 and 11 on the scale). When we remove the 4 participants with high severity scores, the correlation drops (*r* = 0.056, *p* > 0.5).

## Discussion

We established a procedure for developing a Sound-Swapped Video Database for the study of misophonia. The validated SSV database, which includes a refined set of 18 misophonic trigger sounds mapped to their original OVS and alternative PAVS sources, is publicly available (see text footnote 2). We have excluded from the database the two video pairs that had low audio-visual match scores for their PAVS, which indicated that they are not believable sources for the trigger sound. We have also made public the aggregate responses of the sound-video match and pleasantness of the stimuli, as well as detailed instructions for future researchers to develop their own stimuli as needed. It is our hope that as a community of researchers and practitioners, the co-development of these stimuli can encourage more multimodal perspectives in understanding misophonia.

Our studies validated that our SSV database is constructed of videos with a moderate synchronized match between trigger sounds and video sources, and that sounds presented in the PAVS context are perceived as significantly more pleasant than the same sounds presented in the OVS context. We also found that the modulating effect of PAVS is substantial and works across participants regardless of their reported misophonia severity scores. In fact, individuals who reported higher severity scores on the MQ rated the OVS-paired sounds as less pleasant than those with lower severity scores, making the attenuation effect stronger for these individuals. This suggests that future studies could evaluate if the comparison between PAVS and OVS stimuli may have a therapeutic effect for misophonia sufferers if an extension of our previous work (Samermit et al., 2019) were to be applied to participants with misophonia. Those with more severe symptoms may therefore stand to benefit from a dedicated PAVS-based intervention that trains them to associate triggering sounds to non-trigger visual sources. Future research on the short- and long-term effects of sound-swapped videos on misophonic trigger responses should be explored.

Further, we found an order effect that suggests there is learning involved in the perception of misophonic trigger sounds, where the pleasantness ratings for PAVS-paired sounds were lower if the corresponding OVS videos were presented first. This indicates it may be harder to associate a trigger sound with a PAVS once the exact sound has already been heard in the original OVS context. This result is in line with Kumar et al. (2021), where the visual or auditory cue may act as a medium for understanding the action that resulted in the sound. If an individual with misophonia hears a sound and maps it onto the action of someone chewing on chips, and then sees as PAVS attempting to remap it, that representation may have already been learned and difficult to remap. This suggests that for maximum effectiveness, PAVS-paired sounds should be presented first to establish a stronger association, prior to presenting the sound in the OVS context. However, we note that despite the order effect, PAVS-paired sounds still were rated as more pleasant than OVS-paired sounds, even when the OVS-paired sounds were presented first. We also note that in a therapeutic context, PAVS-paired sounds would be presented repeatedly under different circumstances and in different contexts, over an extended period of time. It remains an empirical question whether this intervention will significantly reduce the severity of misophonia symptoms or associated functional impairment.

Another line of work aims to understand intersections between misophonia and other psychiatric disorders or syndromes. Clinical researchers have found that those with misophonia report comorbidities with other psychiatric disorders such as obsessive-compulsive personality disorder, mood disorders, ADHD, and autism spectrum disorder (Jager et al., 2020), post-traumatic stress disorder (PTSD) and a novel audio-visual phenomena called the autonomous sensory meridian response, or ASMR (Rouw and Erfanian, 2018).

Even with these comorbidities, it is unlikely misophonia can be fully explained by an underlying psychological disorder (Schröder et al., 2013; Rouw and Erfanian, 2018). However, the emotional regulation and dysregulation associated with psychiatric disorders have been found to mediate trigger responses (Cassiello-Robbins et al., 2020). Specifically, misophonia has been found to be associated with anxiety, depression, and personality disorder symptoms, with anxiety as a mediator between personality disorder symptoms and misophonia (Cassiello-Robbins et al., 2021). As such, different forms of cognitive behavioral therapy, including transdiagnostic and counterconditioning approaches (Schröder et al., 2017; Lewin et al., 2021), and inhibitory learning approaches (Frank and McKay, 2019) that address emotional responses and contextual factors around a trigger stimulus-response pairing have shown some promising results. As the field continues to learn more about misophonia, it is possible that these clinical approaches may be complemented by the cross-sensory remapping approach we introduced here.

Existing research has already begun exploring how stimuli manipulation can be used in cognitive behavioral therapy (CBT) to alleviate misophonic trigger reactions. Schröder et al. (2017) conducted group CBT sessions with four main therapeutic exercises, including stimuli manipulation and counterconditioning where participants manipulated aspects of their own trigger sounds such as the pitch, duration of sound, and associations with visual stimuli. As part of this exercise, participants combined trigger sounds with pleasant stimuli, and were tasked with decreasing avoidant coping strategies when listening and watching their own stimuli at home. The researchers found that stimulus manipulation “helped to decrease the uncontrollability over misophonic triggers” and that the stimulus-grounded practice resulted in participants “feeling less overwhelmed by misophonic sounds” (Schröder et al., 2017, p. 292). We see our work as complementary to Schröder et al. (2017)’s CBT practice, and believe the Sound-Swapped Video Database provides researchers an opportunity to scale a stimulus-grounded intervention for counterconditioning with larger populations.

Our findings should be considered in light of some limitations in our studies. First, we did not confirm *post hoc* whether participants knew or suspected that they had listened to the same sounds twice. As such, we were unable to confirm whether the order effect we identified was driven by this conscious knowledge or association. Additionally, the study was conducted remotely and we did not include any tasks to standardize the volume of different sounds across participants. Thus we do not know how soft or loud participants set their volume to, whether they changed the volume over the course of the study, or whether they were listening with headphones or on a device’s speaker. As such, future remote studies should consider using a volume check task or request for participants to report their device setup to account for potential variability in sound delivery. Replicating these experiments in the lab under controlled auditory presentation conditions would be important for future research. Finally, by asking participants to attend to the goodness of the match between the sound and video of each clip, we may have caused an ironic effect where mismatches between the audio and video were made more salient, potentially reducing benefits of PAVS.

We also presented participants with decontextualized examples of trigger stimuli: 12 s videos with an unknown actor. Our 12-second clips were produced to be long enough to provide stimuli for researchers conducting psychological or neuroimaging research (e.g., fMRI) on misophonia, but may not be long enough to elicit strong trigger responses. Future stimuli development may also consider developing longer videos, including ones in more naturalistic contexts.

Existing research suggests that misophonic trigger responses are susceptible to contextual factors, such as the meaning tied to the sound, social control, or social relationships (Schröder et al., 2013; Rouw and Erfanian, 2018). Our stimuli lacked social contexts and, more specifically, any necessary or imposed interactions between the participant and the producer of the sound. By stripping the clips of these higher-level contexts, we are unable to make claims on the generalizability of these results to other examples or situations. Future research may benefit from exploring the role of social context and controllability as a factor that may potentially interact with PAVS-based attenuation of misophonic responses.

The evaluation of our stimuli in Studies 1 and 2 was based on a neurotypical population, and we did not screen for participants with misophonia. Even though we observed a range of misophonia sensitivity and severity scores using the Misophonia Questionnaire (Wu et al., 2014) within our sample, we did not include enough participants with high sensitivity or severity scores to examine the robustness of these effects for these individuals. However, 14 of the participants had a severity score of 7 or higher, qualifying them as having clinically significant misophonic reactions. This lends credibility to the potential efficacy of our PAVS as it relates to misophonia: The mean difference for these participants was driven by a lower baseline for their OVS scores, rather than an increase in pleasantness from the PAVS. In ongoing work, we are examining how individuals with misophonia from the 80 interviews conducted in Study 1 respond to sounds presented in the context of OVS and PAVS. This ongoing work, we hope, will help us identify whether cross-modal remapping of misophonic trigger sounds to plausible, positive alternative video sources might be a viable therapeutic method.

## Conclusion

We hope that by releasing our initial set of OVS and PAVS stimuli, along with aggregate ratings and video production methodology, a collaborative effort across multiple research groups can contribute to and refine the database. It is our hope that our work can inspire and encourage broader brainstorming on plausible alternative sources of trigger sounds. Experienced sound and video editors should be able to produce even better matched videos and produce more examples of each trigger sound and PAVS source. Our SSV stimuli and development guide represent a first step in creating a publically available database of audiovisual stimuli for use in Misophonia research.

## Data Availability Statement

The original contributions presented in this study are included in the article/supplementary material and can be found at https://osf.io/3ysfh/, further inquiries can be directed to the corresponding author/s.

## Ethics Statement

The studies involving human participants were reviewed and approved by University of California, Santa Cruz—Office of Research Compliance Administration (IRB). The patients/participants provided their written informed consent to participate in this study. Written informed consent was obtained from the individual(s) for the publication of any potentially identifiable images or data included in this article.

## Author Contributions

PS and ND were co-PIs on the project and developed the concept for the manuscript, supervised the experiments and data analysis, and co-wrote the manuscript. AA, HT, and SS conducted the interviews with 80 individuals with misophonia to identify common trigger categories, with GM, VR, and VH conducting quantitative and qualitative analyses on these interviews to verify the triggers for stimuli development. MY, AK, and CK developed and refined the video construction method, and produced all of the stimuli. MY authored the detailed instruction manual for constructing these stimuli. All authors contributed to the article and approved the submitted version.

## Conflict of Interest

The authors declare that the research was conducted in the absence of any commercial or financial relationships that could be construed as a potential conflict of interest.

## Publisher’s Note

All claims expressed in this article are solely those of the authors and do not necessarily represent those of their affiliated organizations, or those of the publisher, the editors and the reviewers. Any product that may be evaluated in this article, or claim that may be made by its manufacturer, is not guaranteed or endorsed by the publisher.

## Footnotes

1 See https://www.ipr.edu/blogs/sound-design-for-visual-media/foley-sound-effects-sound-design for more information on Foley Sound Design/

2
https://osf.io/3ysfh/
